# The Labyrinth of Pyrexia of Unknown Origin: A Case of Intravascular Diffuse B Cell Lymphoma

**DOI:** 10.4084/MJHID.2013.019

**Published:** 2013-02-20

**Authors:** Sujith V Cherian, Subhraleena Das, Bandita Das Basu, Robert E Hutchison

**Affiliations:** 1 Department of Internal Medicine, SUNY Upstate Medical University, Syracuse NY USA 13210; 2 Department of Internal Medicine, Rabindranath Tagore International Institute of Cardiac Sciences, Kolkata; 3 Department of Pathology, SUNY Upstate Medical University, Syracuse NY USA 13210

## Abstract

Intravascular large B cell lymphoma (IVLBCL) is a rare, aggressive extranodal B cell lymphoma, classified as a subset of diffuse B cell lymphoma. IVLBCL typically occurs in elderly persons and the clinical heterogeneity of the condition makes the diagnosis elusive in most cases. Most of the reported cases have been in the Asian population with the majority of the cases being diagnosed postmortem. We present a unique case of IVLBCL in a 65-year-old Caucasian male who presented with fever of unknown origin.

## Introduction

Intravascular B-cell lymphoma (IVBCL) is a rare and aggressive subtype of extranodal diffuse large B-cell lymphomas. First described by Pflegrand and Tappeiner^1^ in 1959 as “angioendotheliomatosis proliferans systemisata”, it is characterized by preferential growth of neoplastic lymphoma cells within the lumina of small blood vessels, particularly capillaries.^1^

## Case

A 65-year-old Caucasian male was referred to our institution by his primary care physician for evaluation of fever of 3 weeks duration. Review of the systems was positive for the presence of decreased appetite, a weight loss of around 15 pounds and increasing fatigue over the previous 4 weeks. He had no history of recent travel or any exposure to pets. He was sexually active with one partner for the last 30 years. Physical examination did not reveal any evidence of lymphadenopathy or masses. Laboratory data was significant for normal leucocyte count with monocytosis (18%) on differential, calcium level of 11.4 mg/dL (ref range, 9–10.5 mg/dL). Workup for human immunodeficiency virus (HIV), fungal, viral etiologies and endocarditis remained negative. Rheumatology workup, blood, urine cultures, serum/urine protein electrophoresis were negative. Other electrolytes and hepatic function panel was within normal limits. Mild hypercalcemia in the setting of fever of unknown origin warranted further investigation for any underlying malignancy. A lactate dehydrogenase (LDH) level of 1276 IU/L (Ref range 105–250 IU/L) provided a very non specific clue. However, radiological investigations including thoracic and abdominal CT scan revealed no evidence of malignancy or lymphadenopathy, but were significant only for mild splenomegaly. Undeterred by the negative investigations and solely in pursuant of a strong clinical suspicion, the decision to do a bone marrow biopsy was made. The bone marrow biopsy revealed large atypical CD20 positive lymphoid cells in sharply demarcated clusters restricted to the sinusoids, the cells characterized by markedly irregular nuclei and multiple nucleoli, thus clinching the diagnosis of intravascular large B cell lymphoma (Figure 1a, b & c). The patient was subsequently started on chemotherapy with R-CHOP (Rituximab, Cyclophophamide, Adriamycin, Vincristine and Prednisone). Following eight cycles of R-CHOP, the patient has been afebrile and doing well and is considered to be in remission eight months later, with repeat bone marrow biopsy showing disappearance of lymphoma cells (Figure 1d).

## Discussion

Intravascular large B cell lymphoma (IVLBCL) is an extremely rare extra nodal form of Non Hodgkin’s lymphoma (NHL) characterized by its covert presentation and highly aggressive course.^2^ IVLBCL is defined by WHO as an extremely rare and highly aggressive lymphoma variant, identified by the proliferation of large tumor cells within the lumen of small to medium sized vessels,^2^ with approximately 300 cases reported so far. Median age of presentation is 70 years with particularly male predominance.^3^ Two variants have been described based on geographical prevalence: a) Classical variant characterized by neurological and dermatological manifestations, generally seen in the western population and b) Asian variant characterized by hematological manifestations like hemophagocytic syndrome, hepatosplenomegaly, fever and thrombocytopenia, most commonly described in Asian population, particularly Japan.^1^ The Asian variant of IVLBCL, namely with isolated bone marrow involvement in Caucasians is an extremely rare occurrence and has been reported in very few instances.

The cellular origin of IVlBCL remains obscure, but based on the presence of somatic mutations in the immunoglobulin heavy-chain variable region; post-germinal center cells have been postulated to be the origin.^4^ A defect in the adhesion molecules CD 29, CD54 (intercellular adhesion molecule-1) and CD 11a is probably the root of intravascular localization of the tumor cells. The neoplastic cells commonly express B cell antigens CD 19, CD 20 and CD 79a,^1^ however there is no characteristic cytogenetic aberration.^1^ Involvement can be ubiquitous with varied symptomatology. Bone marrow involvement is typically very late in the disease, especially in the classic variant among Caucasians and is generally found in autopsy in 50% of cases. Myriad of clinical manifestations ranging from stroke like symptoms, pyrexia of unknown origin to nephritic syndrome make this rare entity a diagnostic challenge only to be met with a high index of suspicion and an unrelenting thorough workup. The gold standard to diagnose is organ biopsy, which can be guided by FDG-PET/CT scan if clinical, radiological or hematological workups including a bone marrow biopsy continue to remain unrevealing.^5^ Treatment is based on anecdotal experience, with R-CHOP regimen being the most widely used. Prognosis overall remains poor, not only for the aggressiveness of the disease per se but also for the widespread dissemination that happens before the diagnosis is chanced upon.^1^

Our case stands apart, for it uniquely describes the rare occurrence of Asian variant IVLBCL and early bone marrow involvement in a Caucasian at the same time reiterating the importance of considering IVLBCL while dealing with pyrexia of unknown origin. Only with pursuance of our clinical suspicion, we had the fortune of making an early diagnosis which has translated into remission because of timely institution of therapy.

## Figures and Tables

**Figure 1a:**
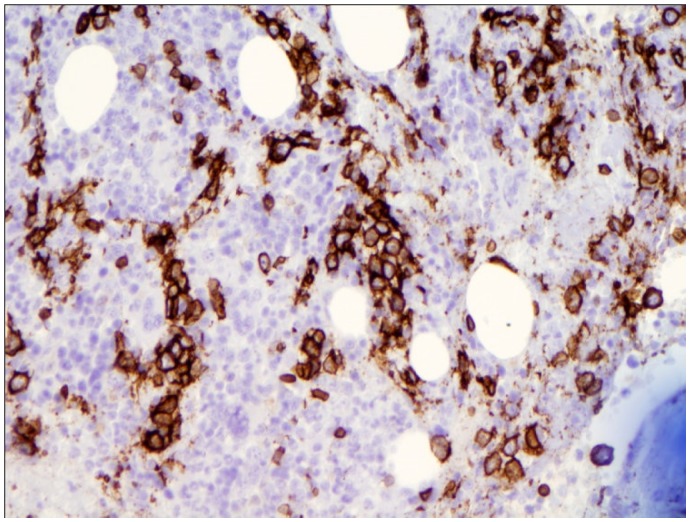
Bone marrow biopsy specimen showing CD 20 positive atypical cells in clusters (original magnification X 200) within sinusoids of bone marrow.

**Figure 1b:**
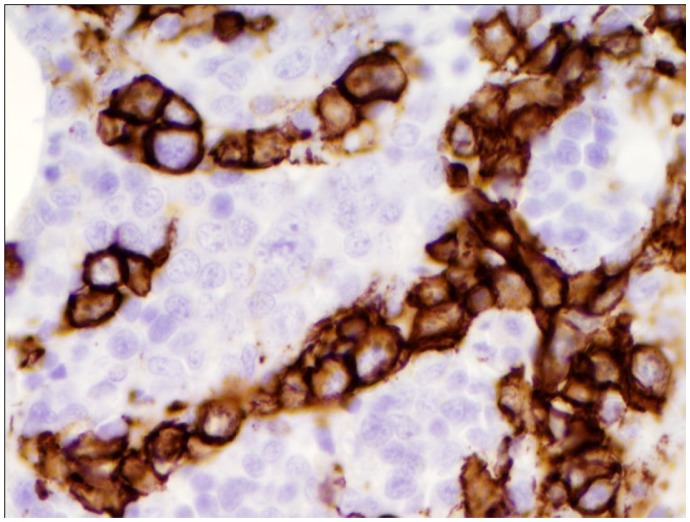
Atypical lympohocytes staining positive with CD20 stain (original magnificationx500) within the sinusoids of the bone marrow.

**Figure 1c:**
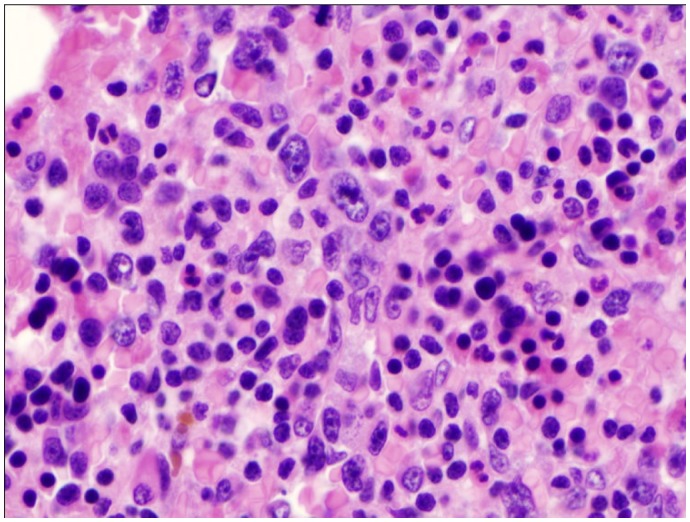
Hematoxylin-eosin stain of bone marrow specimen showing large atypical lymphocytes in clusters within sinusoids.

**Figure 1d:**
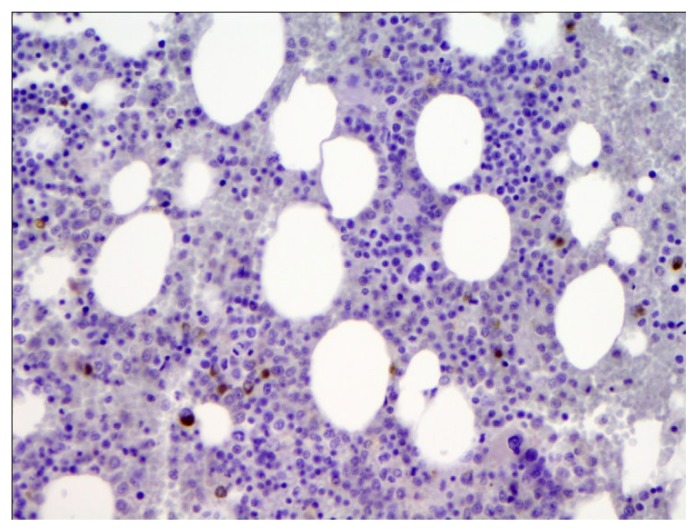
Bone marrow specimen post chemotherapy showing complete disappearance of CD20 positive atypical cells.
